# Optimizing and hyper-tuning machine learning models for the water absorption of eggshell and glass-based cementitious composite

**DOI:** 10.1371/journal.pone.0296494

**Published:** 2024-01-02

**Authors:** Xiqiao Xia

**Affiliations:** 1 College of Mathematics, Sichuan University, Chengdu, Sichuan, China; 2 Teachers College, Columbia University, New York, New York, United States of America; Cardiff Metropolitan University, UNITED KINGDOM

## Abstract

Cementitious composites’ performance degrades in extreme conditions, making it more important to enhance its resilience. To further the adaptability of eco-friendly construction, waste materials are increasingly being repurposed. Cementitious composites deteriorate in both direct and indirect ways due to the facilitation of hostile ion transport by water. The effects of using eggshell and glass powder as partial substitutes for cement and sand in mortar on the water-absorption capacity were investigated using machine learning (ML) modeling techniques such as Gene Expression Programming (GEP) and Multi Expression Programming (MEP). To further assess the importance of inputs, sensitivity analysis and interaction research were carried out. The water absorption property of cementitious composites was precisely estimated by the generated ML models. It was noted that the MEP model, with an R^2^ of 0.90, and the GEP model, with an R^2^ of 0.88, accurately predicted results. The sensitivity analysis revealed that the absorption capacity of the mortar was most affected by the presence of eggshell powder, sand, and glass powder. GEP and MEP model’s significance lies in the fact that they offer one-of-a-kind mathematical formulas that can be applied to the prediction of features in another database. The mathematical models resulting from this study can help scientists and engineers rapidly assess, enhance, and rationalize mixture proportioning. The built models can theoretically compute the water absorption of cement mortar made from eggshell powder and glass powder based on varied input parameters, resulting in cost and time savings.

## 1 Introduction

The trash produced by agriculture and industry is staggering [1]. Substantial economic losses have come from the reckless disposal of hazardous solid wastes, many of which are pathogenic, flammable, highly reactive, and degrading [2]. In addition, modern infrastructure development’s rising need for construction materials contributes to environmental problems and the diminution of natural raw materials [3,4]. Thus, reusing trash to make new building supplies is a viable option [5–7]. In the construction industry, cement-based materials (CBMs) are widely used [8]. Their widespread use can be attributed to many factors, including their inexpensive price, resistance to extreme temperatures, and versatility in form. It is estimated that CBMs are used globally at a rate second only to water [9]. Cement is third in energy usage behind aluminum and steel. Massive volumes of CO_2_ and other greenhouse gases are released into the atmosphere during the production of cement [10]. Due to its high cost, researchers have been looking for cheaper alternatives to cement. CBMs are an example of a resource-efficient building material in which waste materials are substituted for natural aggregates [11].

A common example of farm waste is eggshells. The eggshell accounts for roughly 10% of the entire egg weight. With a growing demand for eggs around the world, the annual production of eggshells has increased to almost 8 million tons [12]. Despite their many uses, eggshells are often thrown away [13]. The calcium-silicate-hydrate (C-S-H) gel employed by CBMs is largely composed of calcium carbonate [14]. In place of sand and cement, it has been proposed that recycled eggshells be utilized to create building materials. Furthermore, a great deal of glass is thrown away, and much of it winds up in landfills. When equated to other waste materials like wood and plastic, glass has a high chemical resistance. Glass is indestructible even after being buried for a long period [15]. The world is currently paying close attention to how to best dispose of glass trash. Recycling glass by melting it down and making new glassware is a widespread practice. However, reprocessing is a lengthy procedure. Glass needs to be collected, cleaned, and melted down before it can be recycled into new items [16]. The aggregate produced from recycled glass can be used in construction. Researchers have discovered that fragments of glass can be used to replace portions of the aggregates and cement in CBMs [17]. Glass can be used to isolate and neutralize potentially harmful constituents of CBMs. Previous research has shown that glass garbage recycling in CBMs is an effective strategy [18]. To lessen the adverse impacts on the environment, CBMs can be made from recycled materials including glass, eggshells, and excess cement and aggregate. The potential of employing eggshell and glass waste as cement or fine aggregate substitutions in CBMs has been tested in experiments [18,19]. To hasten the broad usage of eggshell and glass debris in CBMs, novel ways to evaluate material qualities are urgently needed.

Carbonation, alkali-silica reaction, freeze-thaw shrinkage, sulfate attack, and chloride attack are only some of the deteriorations that might happen to CBM-made structures during the course of their service life [20]. Water has been proven to play a role in all degradation processes, both straight away and indirectly, by providing a conduit for the passage of aggressive ions [21]. Therefore, a comprehensive understanding of water movement in CBMs is required for durability evaluation, service life predictions, and the definition of durability-based design concepts. One of the most important characteristics of any substance is its water absorption (W.A), which indicates how well it can take up and transport water through capillary pores [22]. Gravimetric analysis of W.A. wasn’t used for studying construction materials like stone, brick, and CBMs until 1977, 20 years after it was first used in 1957 for soil science [23]. The rate of W.A was shown to be the determining factor in the amount of degradation caused by the rehydration of calcium oxide during studies on the involvement of W.A in the degradation of CBMs at high temperatures [24]. The W.A approach was used to evaluate Grade M40 pavement concrete, in which wollastonite was used as a major substitute for cement. The W.A test was utilized for the initial absorption, carbonation depth, and compressive strength measurement to assess the concrete cover zone’s open porosity [25]. Furthermore, W.A was utilized in the study of many sorts of concrete, including but not limited to lightweight [26], waste utilization [27–29],recycled aggregate [30], self-curing [31], and fiber-reinforced [32] CBMs. When it comes to forecasting the longevity of CBMs, W.A. has been established as a reliable method [33]. Several ML techniques are being employed to investigate the various properties of construction materials, as provided in Table 1, being an advanced and swift approach.

**Table 1:** List of previous studies conducted using machine learning.

Research Article	Materials studied	Properties predicted	ML method employed
[34]	Fiber-reinforced concrete	Ultrasonic pulse velocity	Gradient boosting (GBR), Extreme gradient boosting (XGB).
[35]	Fly ash concrete	Compressive strength	GEP and Artificial neural network (ANN).
[36]	Nano-silica influence on mortar	Compressive strength	GEP, ANN, and Evolutionary Polynomial Regression (EPR)
[37]	OPC-concrete	Compressive strength	Support vector machine (SVM) and ANN.
[38]	Eggshell powder-based mortar	Mechanical properties	Multilayer perceptron neural network (MLPNN) and XGB.
[39]	Lightweight geopolymer mortar	Compressive strength	GEP
[40]	Metakaolin-based concrete	Mechanical properties	GEP and MEP

In an effort to save costs and save time, specialists are developing performance prediction models for materials and buildings [41–43]. Prediction models are used to estimate attributes, and some of these simulations are based on regression. Model development in this area is currently being led by machine learning (ML) and other forms of artificial intelligence [44–46]. The application of ML techniques to the evaluation of construction materials’ effectiveness has recently garnered more attention. While most recent ML studies have focused on the durability of traditional CBMs [47], only a select few have addressed the prediction of attributes for CBMs modified with eggshells and glass powder [48]. However, no machine learning (ML)-based study was found that predicted the functionality of cement mortar made from eggshells and glass powder.

The intent of this study is to create and evaluate models for the W.A of eggshell and glass powder-based cement mortar (E&GP-CM) using Gene Expression Programming (GEP) and Multi Expression Programming (MEP) and to look at how the input components affect the W.A. The goals of (i) constructing GEP and MEP models using experimental data, (ii) validating the models constructed using statistical measures, and (iii) studying the impact and interaction of input constituents using sensitivity analysis, all contributed to the success of this endeavor. Experimenting with materials is a time- and labor-intensive process due to the need to acquire materials, create samples, wait for them to cure, and then evaluate those using tests. The construction sector could benefit from the application of innovative ML techniques like GEP and MEP to address these problems. It is challenging to experimentally assess the cumulative effect of the many elements that affect the W.A capability of CBMs. Sensitivity analysis is a powerful method for studying the effect and connection between input variables and CBMs’ W.A. Data for GEP and MEP model building and sensitivity analysis could come from either existing literature or new experimental work. So, the collected data can be used for things like running GEP and MEP algorithms, making material property estimates, and studying the impact of input variables. To evaluate the efficacy of ML methods in predicting the W.A of E&GP-CM, this study used an experimental dataset with 234 points and 7 variables as inputs. Also, raw materials’ importance and impact on the W.A was determined by sensitivity analysis and interaction study.

## 2 Research methods

### 2.1 Data development and analysis

**Fig 1:**
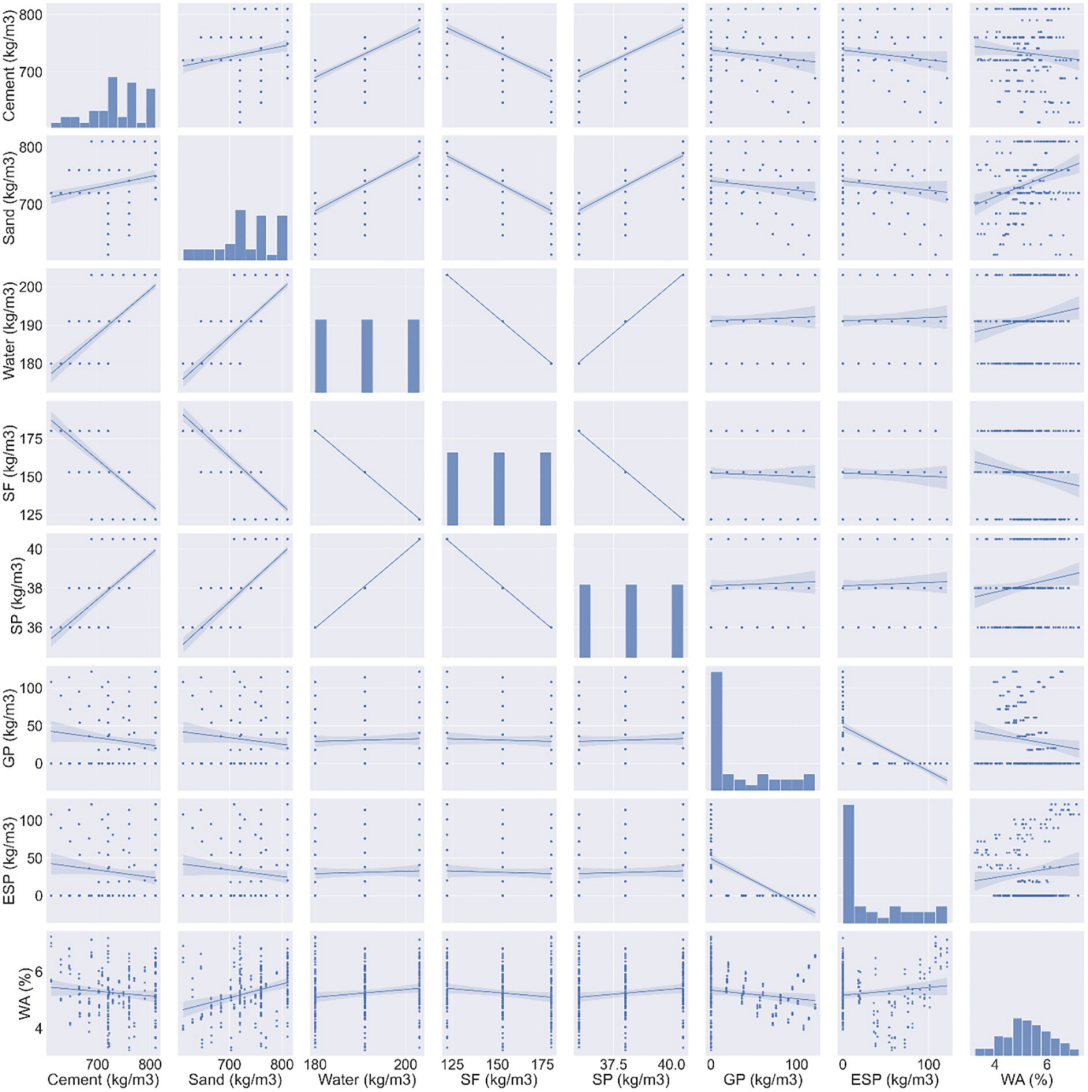
Distribution of database input and output characteristics in terms of frequency.

This work used a dataset consisting of 234 data points from an experimental investigation to run GEP and MEP models to predict the W.A of E&GP-CM [49,50]. The work presented here predicts the W.A of E&GP-CM based on seven input variables: cement (C), water (W), sand (S), silica fume (SF), superplasticizer (SP), recycled glass powder (GP), and eggshell powder (EP). GEP and MEP, both machine learning approaches, were used to make predictions about the W.A of E&GP-CM. Data preparation was used to collect and organize the data. Preparing data for data mining is one method for tackling a problem with the well-known knowledge discovery method. The goal of data preparation is to simplify the data by removing noise and other extraneous information. In addition, experts from a wide range of disciplines have speculated that the ratio of data points to inputs is crucial to the performance of the suggested model. The optimal model requires a ratio greater than 5, allowing for the testing of data points to determine the link between the selected variables [51]. The current study uses seven inputs to forecast the W.A of E&GP-CM, and the resulting ratio of 33.4 satisfies the standards set out by the researchers. The model was examined by means of regression and techniques for distributing errors. Descriptive statistics were run on these data, and the results are shown in Table 2. Descriptive statistics are used to summarize a study’s data and reveal its most salient characteristics. The sample and the metrics are summed up in a few easy sentences. Basic graphical analysis and statistical tests are the bedrock of any quantitative data investigation. The accuracy of the utilized models has also been assessed using the validation technique. The relative frequency distribution of all the variables is shown in the histograms in Fig 1. The overall frequency distribution of a data set can be described by its individual input variables’ distributions. By generating a relative frequency distribution, it is easy to see how frequently values appear in a dataset.

**Table 2:** Variable descriptions based on statistics.

Descriptive statistics	Cement	Sand	Water	Silica fume	Superplasticizer	Glass powder	Eggshell powder	W.A
Unit	(kg/m^3^)							(%)
Mean	732.51	735.62	191.33	151.67	38.17	30.83	30.83	5.25
Standard Error	3.50	3.56	0.62	1.55	0.12	2.62	2.62	0.06
Median	722.00	729.00	191.00	153.00	38.00	0.00	0.00	5.19
Mode	810.00	810.00	203.00	122.00	40.50	0.00	0.00	5.05
Standard Deviation	53.51	54.39	9.41	23.75	1.84	40.15	40.15	0.89
Sample Variance	2863.15	2958.18	88.60	563.97	3.40	1611.73	1611.73	0.79
Kurtosis	-0.61	-0.61	-1.51	-1.51	-1.51	-0.61	-0.61	-0.48
Skewness	-0.24	-0.32	0.05	-0.08	0.14	0.93	0.93	-0.03
Minimum	612.00	612.00	180.00	122.00	36.00	0.00	0.00	3.22
Maximum	810.00	810.00	203.00	180.00	40.50	121.50	121.50	7.23

### 2.2 Models’ development

To get the desired result from ML techniques, a high number of parameters for input are required [52]. Parameters in the data sample need to be dynamic for an ML technique to produce desirable results; using a fixed value or one with restricted change may produce inadequate outcomes [53]. The first step in developing an AI model is settling on appropriate input parameters. Seven input factors were selected for this investigation because of their potential to have a major impact on the W.A of E&GP-CM. Only 30% of the data was used for actual testing, whereas 70% was used for really training the algorithms.

GeneXproTools version 5.0 was used by GEP to build these models. In order to generate code, GeneXproTools first sorts the variables into categories and then uses randomization and processing to fill in the blanks in the data. The replica that was created is more operative and of greater quality than the original [54]. It supports multiple programming languages, including MATLAB, C++, and Visual Basic, for program generation and model construction. The GEP factors used in this research were determined after extensive testing and a review of related literature. To gauge how various GEP parameters affect prediction accuracy, the initial optimal combination was chosen through trial and error. Each dataset and model in machine learning model training requires a unique set of hyperparameters. These can only be determined through a series of experiments in which different values of the model’s hyperparameters are tested [55]. As a result, the best possible order of GEP hyper-parameters was chosen and used in the modeling to generate estimates about the outcome and convert those forecasts into easy-to-understand mathematical expressions (see Table 3). The complexity of the model varies with factors such as the number of chromosomes, the number of genes, and the size of the head. The time needed to run the program will grow as the parameters grow larger. However, a more accurate overall model can be achieved with a larger number of chromosomes and genes. Earlier research on E&GP-CM [56] contrasted individual and ensemble machine-learning techniques. This research, however, contrasts the outcomes of two separate genetic ML methods (GEP and MEP). Table 3 displays a comparison of the findings of the current study based on how well their hyperparameters were tuned, how well their statistical checks went, and how well their mathematical expression for future prediction was formed. In addition, the prior study did not provide any guidance on how to tune hyper-parameters for the purpose of developing a mathematical equation for GEP. Unless the Python code is made available, it is also impossible to independently verify the XGBoost method’s efficacy. Two genetic mathematical models are used to present the findings of this investigation. A 70% training set and a 30% testing set were used to create these models. Using all of the data from this investigation, one can confidently make projections about the future.

**Table 3:** GEP and MEP models with set parameters.

MEP		GEP	
Parameters	Settings	Parameters	Settings
Number of sub-populations	50	General	W.A
Sub-population size	100	Chromosomes	200
Code length	40	Genes	4
Cross over probability	0.9	Head size	8
Operators/variables	0.5	Linking function	Addition
Function set	+, —, x, ÷, √	Function set	+, —, x, ÷, √
Number of generations	500	Constant per gene	10
Terminal set	Problem Input	Data type	Floating number
Mutation probability	0.01	Lower bound	-10
Replication number	15	Upper bound	10
Error	MSE, MAE	Mutation rate	0.00138
Problem type	Regression	Inversion rate	0.00546
Number of runs	15	Leaf mutation	0.00546
Number of treads	2	Stumbling mutation	0.00141
		IS transposition rate	0.00546
		RIS transposition rate	0.00546
		One-point recombination rate	0.00277
		Two-point recombination rate	0.00277
		Gene recombination rate	0.00277
		Gene transposition rate	0.00277
		Random chromosomes	0.0026

**Fig 2:**
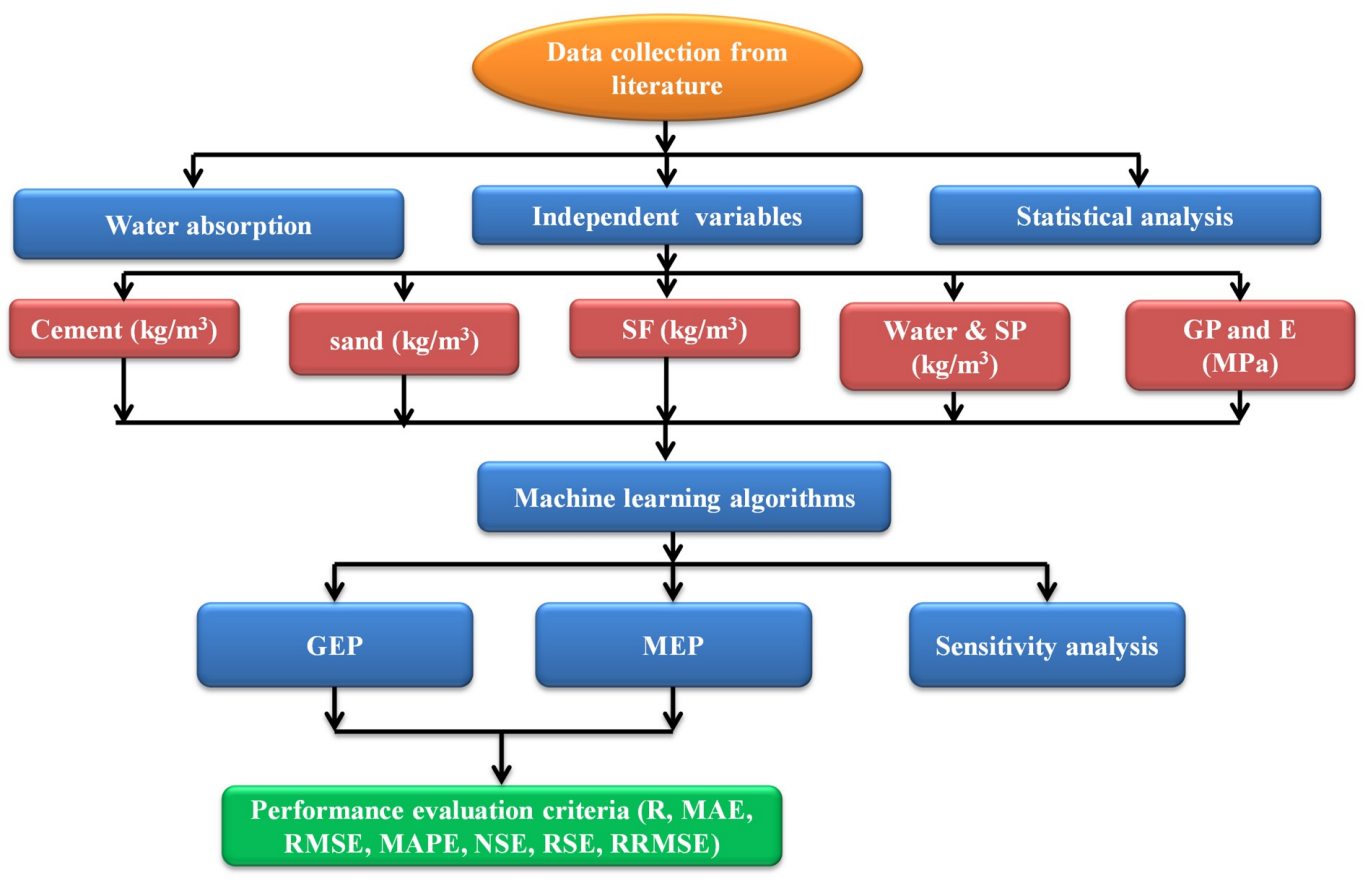
Procedures for creating data samples, fitting models, and validating the results.

As was previously noted [57], the most well-known type of genetic programming, MEP, is the linear variant. With the MEP’s ability to generate a mathematical equation from the developed approximation models [58], an existing collection of 234 datasets was used in the MEPX Version 2023.4.3.0-beta to establish the compaction constraints. The MEP genes are substrings of varying lengths that keep the number of genes per chromosome from changing. Each gene stores an abstract representation of a function or an endpoint, and the genes that code for functions additionally store pointers to the functions’ arguments. Indicator values for the function’s parameters have consistently lagged behind those for the chromosomal location of the relevant function [59]. The process of formula creation is briefly covered, and readers interested in discovering more can find references to the enhanced GEP approach or MEP modeling in the current literature [60]. The hyper-parameters employed to create the MEP model are shown in Table 3. Fig 2 is a flowchart detailing the steps involved in ML-based modeling. The ML algorithms and validation techniques put to use in this research are detailed below.

Both the GEP and MEP benefit from being able to operate within a fixed range of seven input parameters. Because of this quality, you may rest assured that the results you get from using EP and GP as a fine aggregate or cement alternative in CBCs are accurate. Due to their uniform testing procedure and use of identical unit measures, the models produce consistent predictions. Understanding the mix design and the impact of each input parameter is greatly aided by the mathematical equations offered by the models. However, if more than the standard seven parameters are used in the composite analysis, the accuracy of the predicted models may suffer. The created models were built to accept a certain set of inputs and may not perform optimally when given novel data. Changes or inconsistencies in the units of the input parameters might also cause the predictive models to produce inaccurate results. Models’ credibility depends on the consistency of their units of measurement.

#### 2.2.1 GEP method

The genetic algorithm (GA) was created by J. H. Holland and is grounded in Darwin’s theory of evolution [61]. The progression of the genetic process is represented by a series of GAs, and its resolution is shown by chromosomes of constant length. Koza proposed a variant of GA he called gene programming (GP) [62]. GP is a problem-solving technique that is not limited to a certain domain; it generates a genetically evolved model automatically. Because nonlinear structures like parse trees are used in place of fixed-length binary strings, GP is a versatile technique. In accordance with Darwin’s Theory [63], established machine intelligence software handles reproduction-related concerns by relying on certainly performing genomic factors (such as procreation, hybridization, and mutation). In GP, a strategy is developed to remove the worst-performing programs during reproduction. The trees with the lowest fitness are eliminated, as in the preceding example, and the survivors are employed to repopulate the area in accordance with the selected procedure. However, the evolution process safeguards the model’s early convergence [63]. Critical input and measurement sets, core domain operations, fitness evaluation, primary functionality operators (population size, crossover, etc.), and results defined by method-specific termination criteria are the five main parameters that must be specified when using the GP technique [63]. While GP does construct a model automatically [48], the bulk of the parse tree creation is handled by a crossover genomic processor. Complex expressions for desirable features also result from the need for nonlinear GP forms to function as both genotype and phenotype [64].

Ferreira is credited with being the first to introduce GEP, a version of GP [64]. GEP is based on the population generation hypothesis, where analysis of trees is combined with rectilinear chromosomes of a predetermined length in the modeling process. GEP is an improved version of GP that encrypts modest-sized software with the use of simple, fixed-length chromosomes. The use of GEP has the advantage of allowing for the development of mathematical equations that can reliably foretell multipart and nonlinear problems [65]. As in GP, the values for the fitness function, the final set, and the end conditions are all specified. After being identified as chromosomes via the "Karva" language, chromosomes with arbitrary numbers are generated during the execution of the GEP algorithm. A line of fixed length is used in GEP. However, while processing data in code, GP takes into account analysis trees of variable lengths. These separate cords, which are at the outset predetermined as static-length genomes, are then described as nonlinear expression/ analysis trees with separated shapes of varying sizes, representing chromosomes [63]. These genotypes and some varieties of phenol are also encoded separately [64]. The straight transmission of the genome from one generation to the next, without any structural mutations or duplications, is an advantage of GEP.

Normal chromosomes consist of the "head" and "tail" of a chromosome. Thus, the emergence of multi-gene organisms from a single chromosome is a remarkable phenomenon [63]. There are logical operations, mathematical operations, arithmetic operations, and Boolean logic operations encoded in these genes. The genetic code contains operators that connect cells to their designated roles. Karva, a novel language, retrieves and infers the information stored in these chromosomes to create empirical formulas. The expression tree (ET) is then traversed in a leading revolution beginning with Karva. ET stores the nodes in the bottommost layer in line with Eq (1) [65]. Depending on the total number of ETs, the GEP gene’s expression range and the duration of K-expression could differ.


$$ET\;GEP=log(i-\frac{3}{j})$$ (1)

**Fig 3:**
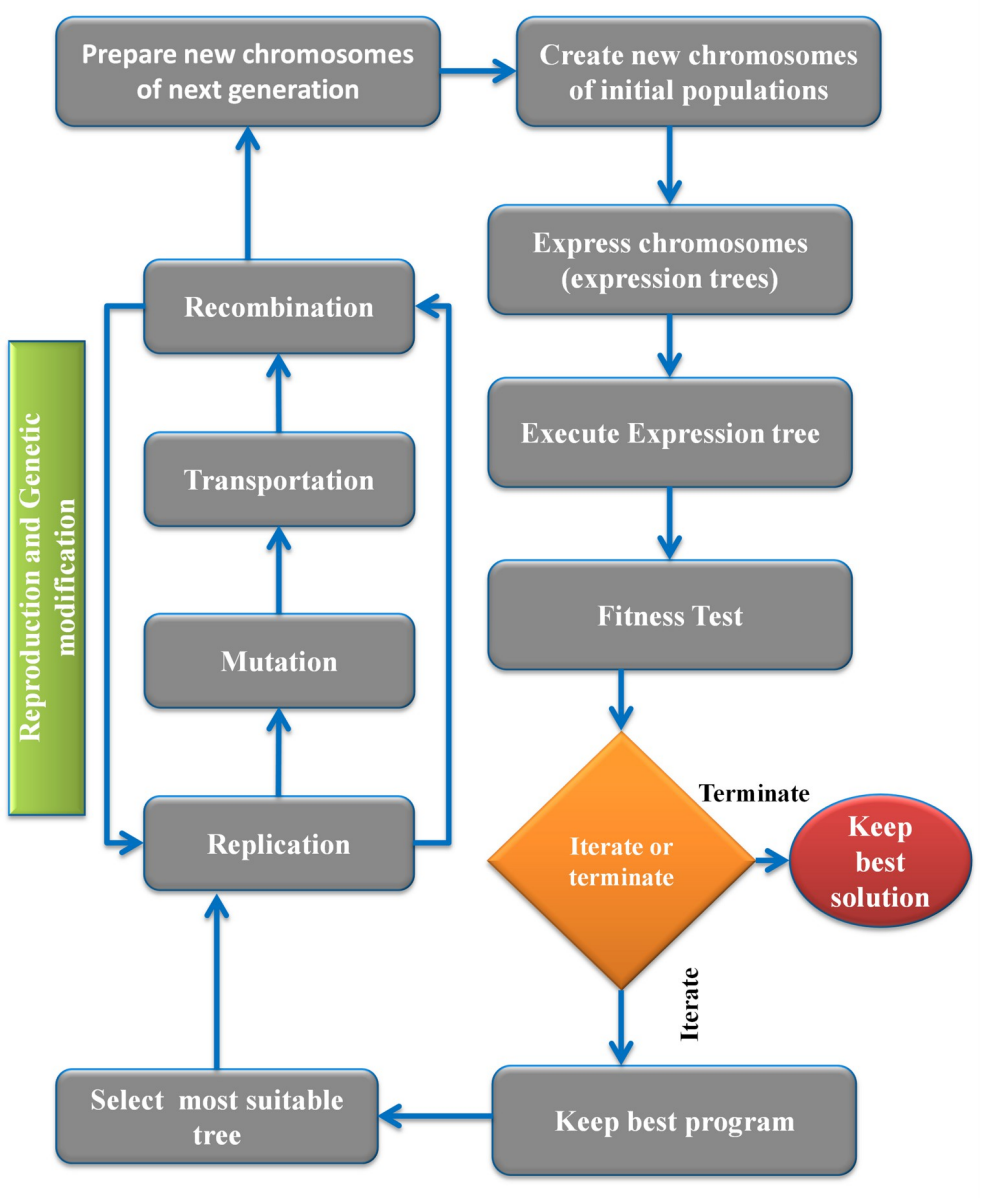
Method flowchart for the GEP procedure [66].

GEP is a complex ML technique because its results don’t rely on any prior connections. Detailed in Fig 3 are the many steps involved in the birth and maturation of mathematical equations in GEP. Each person is born with a set number of chromosomes. When everyone’s health is evaluated when these chromosomes have been confirmed as ETs. The fittest member of the population is chosen to reproduce. The best possible answer is found through an iterative process, including the most suitable persons. Three genetic processes—mutation, crossing, and breeding—are ultimately employed to arrive at the ultimate numerical expression.

#### 2.2.2 MEP method

**Fig 4:**
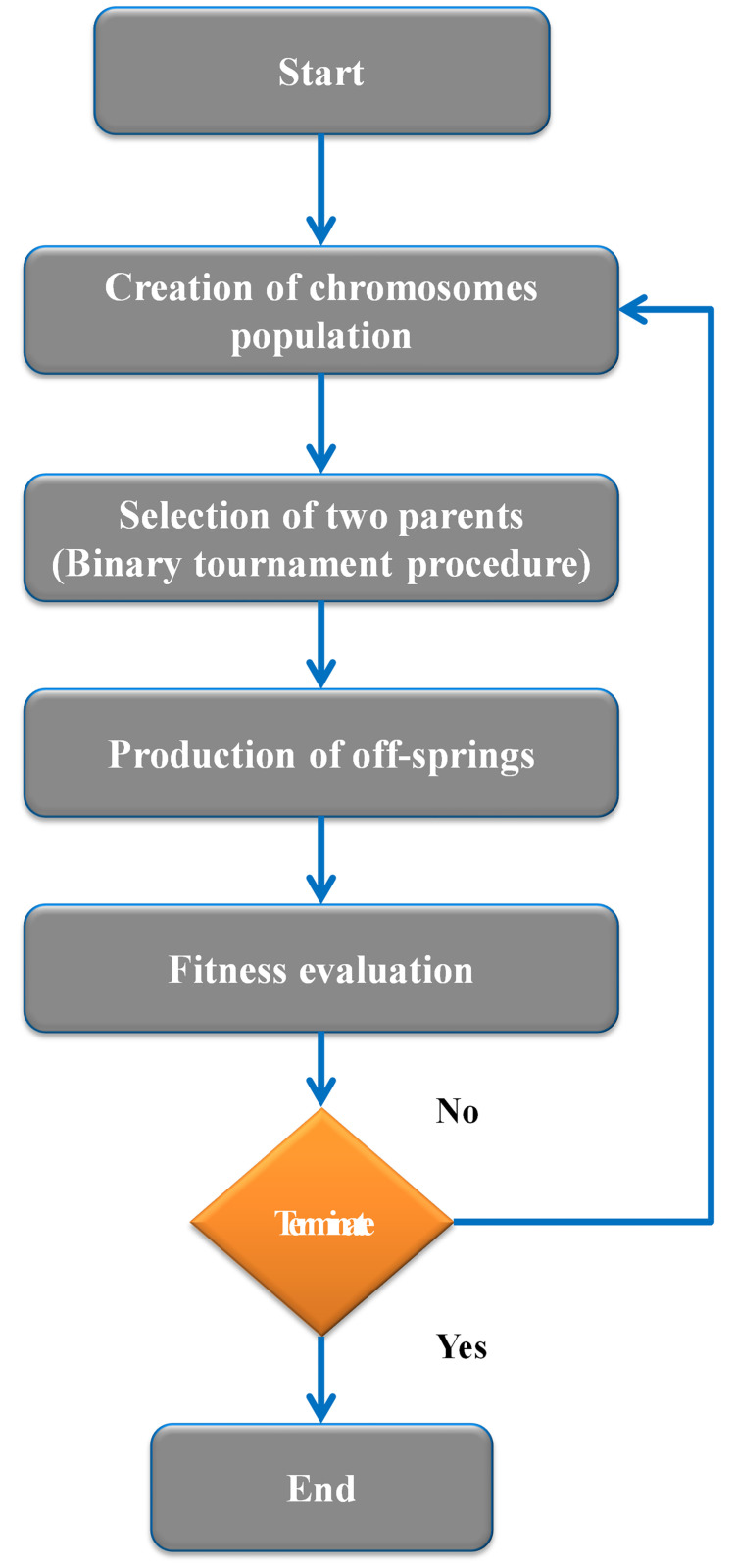
Method flowchart for the MEP procedure [66].

The MEP uses linear chromosomes, making it an advanced and illustrative linear-based GP approach. The core MEP System is very close to the core GEP System. The ability to encode many pieces of software (alternatives) into a single chromosome is a defining feature of MEP, a relatively modern offshoot of the GP approach. The best chromosome is then chosen by measuring fitness [60], yielding the ultimate solution. The process of a twofold environment being recombined into dual unique descendants, according to Oltean and Grosan [59], leads to the assortment of two parents. As can be seen in Fig 4, the process keeps going till the optimal package is found prior to the termination criterion. This is the site where newborn mutations take place. The MEP approach permits the suitability of several factors, much like the GEP model. Multi-expression programming is governed by a number of criteria, such as the number and extent of subpopulations, the length of the algorithm/code, the probability of crossover, and the set of functions [67]. When the population extent is the total number of packages, assessing the population is more difficult, and taking the population size into account is more time-consuming. The size of the generated mathematical expressions is also significantly affected by the length of the code. The amount of MEP parameters used to create a reliable model of W.A is shown in Table 3.

The evaluation and modeling steps of both methods often make use of literature data sets [57]. Some researchers believe that the tremendously prevalent linear GP approaches, including the MEP and GEP approaches, can more reliably predict the qualities of sustainable concrete. Grosan and Abraham [68] found that when compared to other neural network-based approaches, the combination of linear genetic programming (LGP) with MEP yielded the best results. The method by which the GEP actually operates is somewhat more intricate than that of the MEP [67]. Despite the fact that MEP is less compact than GEP [58], it varies in that (i) code can be re-utilized in MEP, (ii) non-coding sections need not be displayed at a specific position within the chromosomes, and (iii) function argument pointers are plainly encoded in the MEP. The GEP is believed to have superior functionality because it possesses the "head" and "tail" of a distinctive GEP chromosome, mutually of which are loaded with ciphers that efficiently encrypt syntactically correct software programs [59]. This means that each of these genetic methods for engineering problems needs to be evaluated and assessed in greater depth.

### 2.3 Models’ assessment criteria

The arithmetical efficacy of the models developed by GEP and MEP was evaluated across testing set. Seven statistical measures were calculated: Pearson’s correlation coefficient (R), mean absolute error (MAE), root mean square error (RMSE), mean absolute percentage error (MAPE), relative squared error (RSE), Nash-Sutcliffe efficiency (NSE), and relative root mean square error (RRMSE) [57,69–71]. The equations for these statistical measures are given in Eqs 2–8.


$$R=\frac{\sum_{i=1}^{n}\left(a_{i}-{\bar{a}}_{i})(p_{i}-\bar{p_{i}}\right)}{\sqrt{\sum_{i=1}^{n}{\left(a_{i}-\bar{a_{i}}\right)}^{2}}\sum_{i=1}^{n}{\left(p_{i}-{\bar{p}}_{i}\right)}^{2}}$$ (2)

$$MAE=\frac{1}{n}\sum_{i=1}^{n}\left|p_{i}-a_{i}\right|$$ (3)

$$RMSE=\sqrt{\sum \frac{{\left(p_{i}-a_{i}\right)}^{2}}{n}},$$ (4)

$$MAPE=\frac{100\%}{n}\sum_{i=1}^{n}\frac{\left|p_{i}-ai\right|}{T_{i}},$$ (5)

$$RSE=\frac{\sum_{i=1}^{n}{\left(a_{i}-p_{i}\right)}^{2}}{\sum_{i=1}^{n}{\left(\bar{a}-a_{i}\right)}^{2}}$$ (6)

$$NSE=1-\;\frac{\sum_{i=1}^{n}{\left(a_{i}-p_{i}\right)}^{2}}{\sum_{i=1}^{n}{\left(a_{i}-\bar{p_{i}}\right)}^{2}}$$ (7)

$$RRMSE=\frac{1}{\left|\bar{a}\right|}\sqrt{\frac{\sum_{1=1}^{n}{\left(a_{i}-p_{i}\right)}^{2}}{n}}$$ (8)

The ith real and estimated outcomes are denoted by *a*_*i*_ and *p*_*i*_, respectively; *a*_*i*_ and *p*_*i*_, represent the average values of the real and estimated results, respectively, and n represents the total number of observations in the dataset. The effectiveness of a model (ai and pi) is typically quantified using the performance measure R, which represents the comparative association between the expected and actual results. When R exceeds 0.8, there is a robust relationship between the observed and predicted quantities of output [72]. However, it has been found that division and multiplication have no effect on component R. Given its high efficiency and reasonable approximation results, R^2^ was computed between the observed and anticipated data. Higher R^2^ values, near 1, indicate the constructed model is more effective [73]. In a similar vein, both MAE and RMSE handled larger error levels competently. Errors have less of an effect, and the constructed model works better when the MAE and RMSE are closer to zero, as shown in [74]. It is well known that MAE is most useful for continuous and homogeneous data sets. Lower numbers for the errors calculated up top indicate better overall model performance.

## 3 Results and analysis

### 3.1 GEP model

**Fig 5:**
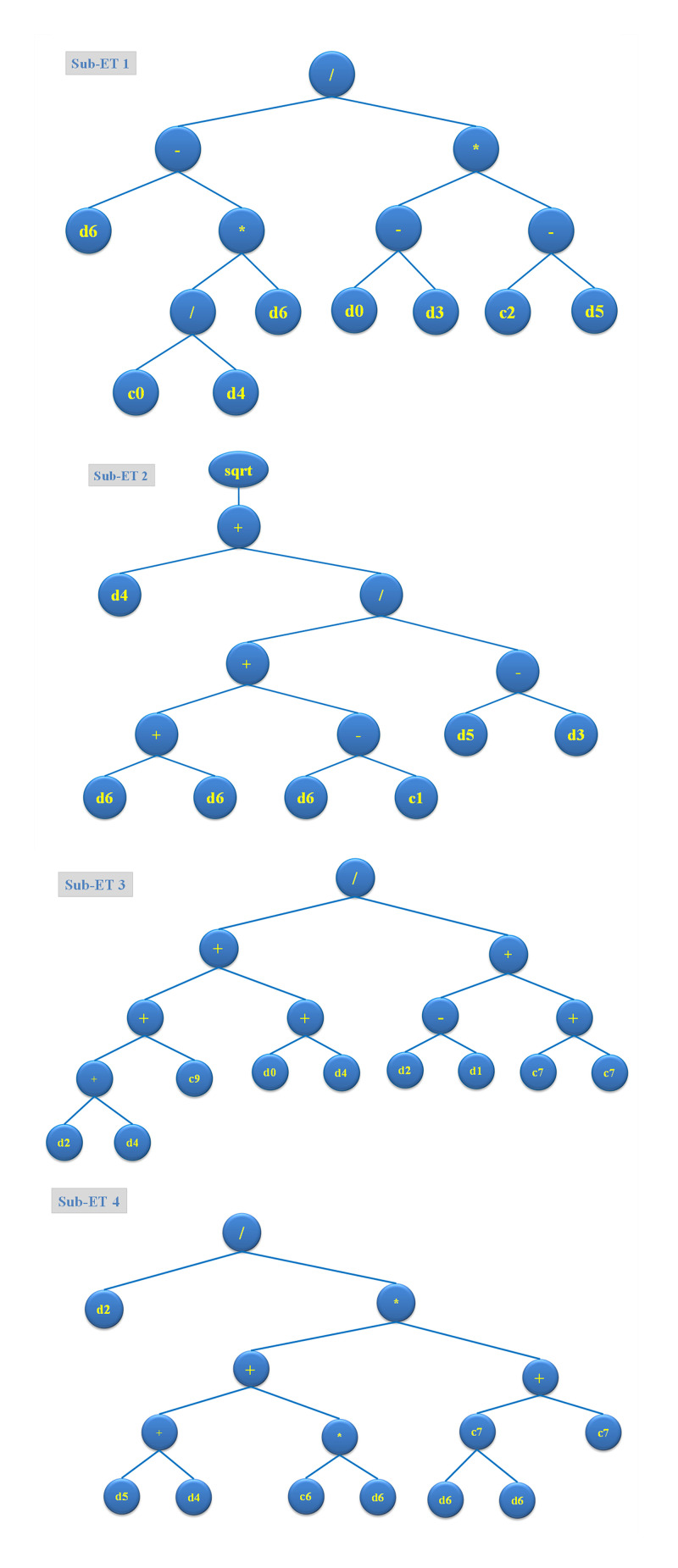
Developed model’s expression tree diagram.

**Fig 6:**
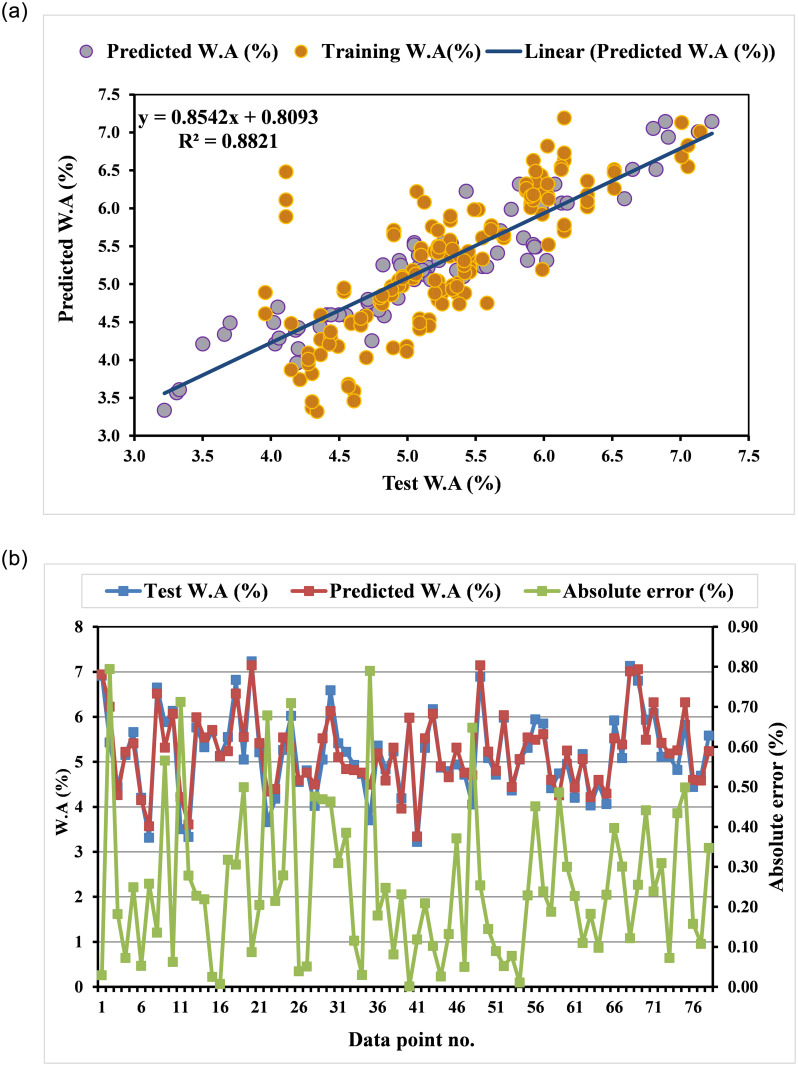
GEP-E&GP model: (a) link among test and predicted W.A; (b) dispersal of test and predicted CS and errors.

The GEP technique, as shown in Fig 5, developed ETs-based models based on head size and chromosome count in order to infer mathematical correlations for determining the W.A. Most of the W.A of E&GP-CM’s sub-ETs are formulated using the five operators of arithmetic (+, -, x, and square root). The GEP model’s sub-ETs are encrypted, producing a mathematical formula that acts as the output. The output value, as shown by Eq 9, can be utilized to predict future W.A of E&GP-CM based on the provided input parameters. For E&GP-CM, the constructed model has sufficient data points and outperforms a perfect model under ideal conditions. The expected outcomes of the model and the experimental values from the testing datasets are compared using regression lines for W.A in Fig 6(a). With an R^2^ of 0.88, suggesting reasonable agreement between the actual and estimated results, the GEP technique was found considerably effective for approximating the W.A of E&GP-CM. Absolute error and experimental values are plotted in Fig 6(b) to provide an idea of how far off the GEP model can be from the truth. Experimental data and the outcomes predicted by the GEP equation are practically identical, with a lowest absolute error of 0.0006% and a maximum absolute error of 0.79%, with an average absolute error of 0.25%. In addition, 47 of the error values were found to be less than 0.25 percent, 24 were found to be between 0.25 and 0.5 percent, and 7 were found to be greater than 0.5 percent. It’s crucial to remember that maximal error frequencies actually occur very infrequently in the case of GEP, as observed in the prior studies [66].

$$\begin{array}{c} W.A\;\left(\%\right)=\frac{EP-\left(\frac{{EP}^{2}}{SP}\right)}{\left(C-W)(-0.173-GP\right)}+\sqrt{\frac{\left(SP+(3EP-5.925\right)}{\left(GP-SF\right)}}\\ +\frac{\left(W+C+2SP+15.303\right)}{\left(W-S-13.480\right)}+\frac{W}{\left(GP+SP-3.562{EP}^{2}-4.614E\right)} \end{array}$$ (9)

Where, W.A: water absorption, C: cement, S: sand, W: water, SP: superplasticizer, SF: silica fume, GP: glass powder, and EP: eggshell powder.

### 3.2 MEP model

An empirical formula was obtained to find the W.A of E&GP-CM by deciphering the MEP outcomes in order to take into consideration the effects of the seven independent components. The final modeled mathematical equations are shown in Eq 10.

$$W.A\;\left(\%\right)=\left(\sqrt{\frac{\left(S-SP+2EP\right)}{\left(\sqrt{C+EP}\right)}}\right)-\left(\frac{\left(2GP-W\right)}{\left(W-EP*SF\right)}\right)$$ (10)

Where, W.A: water absorption, C: cement, S: sand, W: water, SP: superplasticizer, SF: silica fume, GP: glass powder, and EP: eggshell powder.

**Fig 7:**
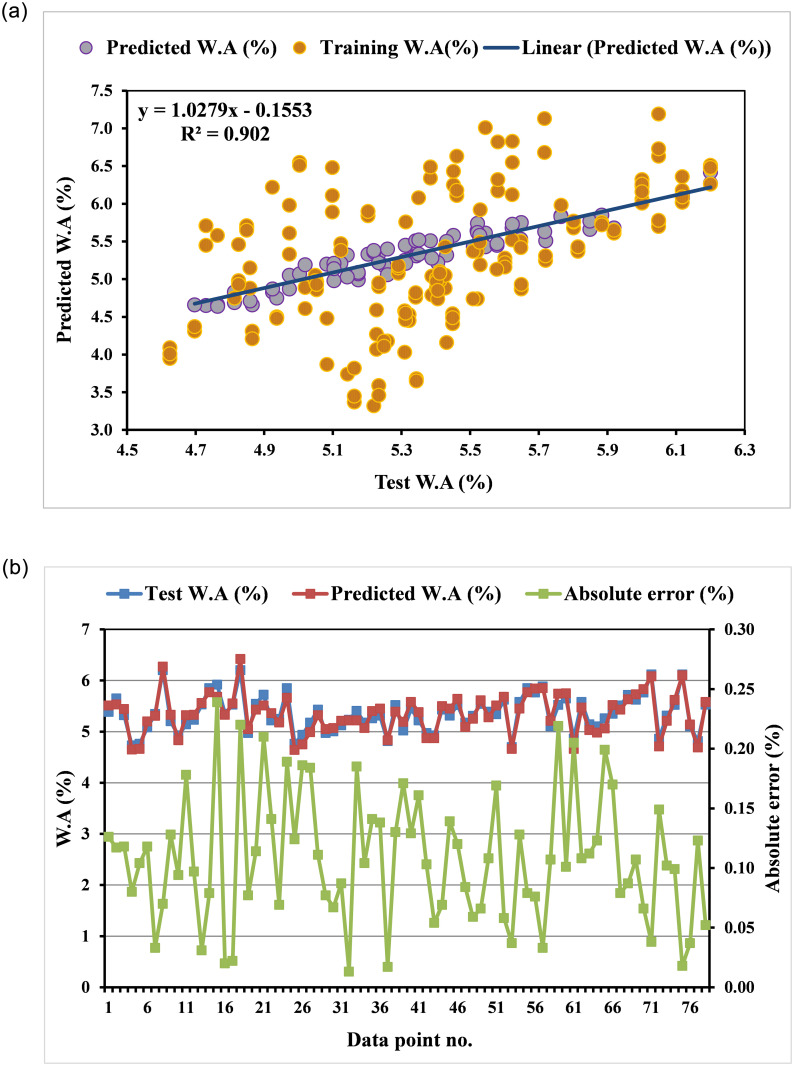
MEP-E&GP model: (a) link among test and predicted W.A; (b) dispersal of test and predicted CS and errors.

The MEP model’s expected and actual results are compared in a similar fashion in Fig 10. The ideal situation is one in which the grade of the regression line is equal to 1. As can be shown in Fig 7(a), the MEP model performed well on testing data, with an R^2^ of 0.90, indicating that it is well-trained and able to manage oversimplification, as well as performing favorably on novel, untested data. The MEP model appears to be more accurate than the GEP model, as indicated by the higher R^2^ value. Observed values and desired values are compared in MEP simulations, and the absolute differences are plotted in Fig 7(b). According to the given information, the margin of error for the MEP forecasts ranged from 0.013 percentage points to 0.239 percentage points. Keep in mind that the maximum error in the MEP projected outcome is significantly lower than the highest error in the GEP model. Strong predictive ability may be found in both the MEP and GEP approaches. The MEP equation reduces the standard deviation of the error as well as the coefficient of correlation. The MEP equation is widely applicable because of its simplicity and compactness. The values of the various statistical errors for both models are also shown in Table 4. The MEP model looks to be superior to the GEP model due to its lower error levels and greater correlation coefficient, similar to the prior studies [75]. It can be argued further that MEP is superior to GEP in estimating the W.A of E&GP-CM from any conceivable perspective. The MEP model’s transparency and clarity are two of its best qualities. To account for the multiplicative impacts of the individual components on the W.A of mortar, MEP uses an equation. This equation is practical since it can be quickly grasped and put to use. The GEP model, on the other hand, is founded on a sophisticated nonlinear equation developed in genetics. The equation’s complexity may make it hard to understand, and it may not shed light on the connections between the variables.

**Table 4:** Indicators of MEP and GEP model performance based on statistics.

Parameters	MEP	GEP
MAE (%)	0.107	0.253
MAPE (%)	3.30	5.20
RMSE (%)	0.120	0.319
NSE	0.922	0.884
R	0.950	0.939
RSE (%)	0.187	0.224
RRMSE (%)	0.122	0.559

### 3.3 Statistical validation of the models

**Fig 8:**
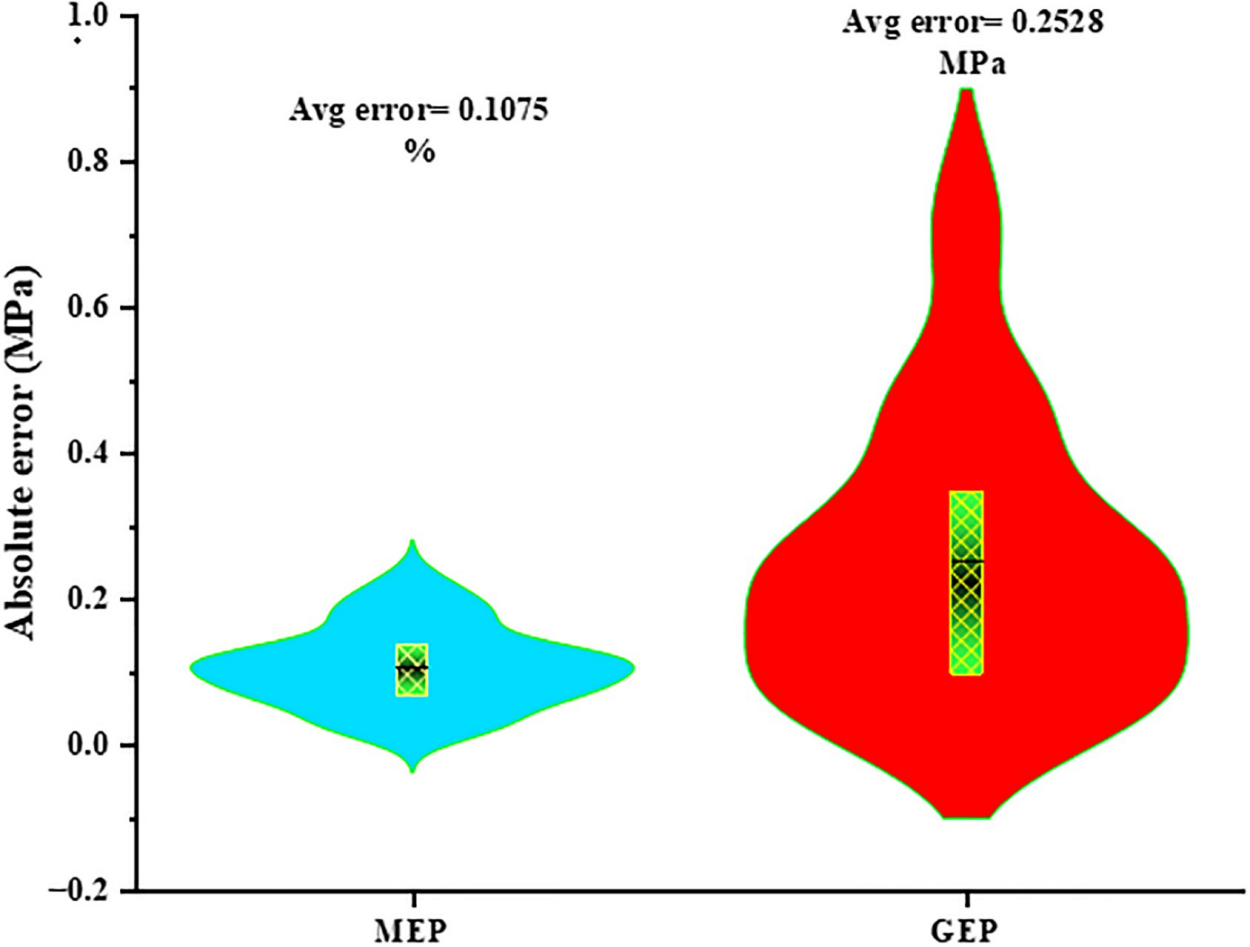
Errors in machine learning models plotted as violin graphs.

The statistical accuracy of the MEP model should also be taken into account. With an impressive R^2^ of 0.90, the MEP model provides a plausible explanation for 90% of the observed W.A of E&GP-CM. The greater R^2^ value achieved by the MEP model during validation also suggests its potential for application in forecasting future data. The high R^2^ result specifies a robust association between the sovereign variables (water, superplasticizer, silica fume, sand, glass powder, cement, and eggshell powder) and the dependent variable (W.A). Additionally, as indicated by a reduced root mean square error (RMSE), the MEP approach showed greater prediction precision than the GEP approach. When the RMSE, MAE, MAPE, RRMSE, and RSE values are smaller, the MEP model’s projected values are more in line with the observed values of the W.A of the mortar samples. Table 4 shows that the statistical metrics RMSE, MAE, MAPE, RRMSE, and RSE, all of which measure the accuracy of a model’s prediction, are much smaller for the MEP models than they are for the GEP model. Table 4 shows that the MEP model has a lower NSE value than GEP, indicating that MEP is more accurate than GEP when it comes to making predictions. NSE is used to evaluate the forecasting skill of the models. The accuracy and foresight of the MEP model can be measured with these parameters. Errors in MEP and GEP models can be seen using violin graphs, as shown in Fig 8. From GEP to MEP, model errors have been reduced. A violin plot is a cross between a box plot and a kernel density plot that emphasizes extremes. It is a tool for representing numerical data graphically. Violin plots exhibit both summary statistics and the density of each variable, whereas box plots can only show summary statistics. Fig 8 shows that the MEP models have a substantially lower peak and general distribution of errors compared to the GEP models, verifying their superior prediction accuracy.

For estimating the W.A of E&GP-CM, the MEP method is recommended because of its simplicity, arithmetical performance, and capacity to integrate the effects of glass powder and eggshell powder in a linear equation. These outcomes may have real-world insinuations for figuring out how to optimize the components of E&GP-CM for the required W.A in construction projects. These results pave the way for more precise forecasting models for various types of adapted mortar/concrete, which in turn allows for more efficient and ecologically responsible building practices.

### 3.4 Impact and interaction of inputs

#### 3.4.1 Sensitivity analysis

**Fig 9:**
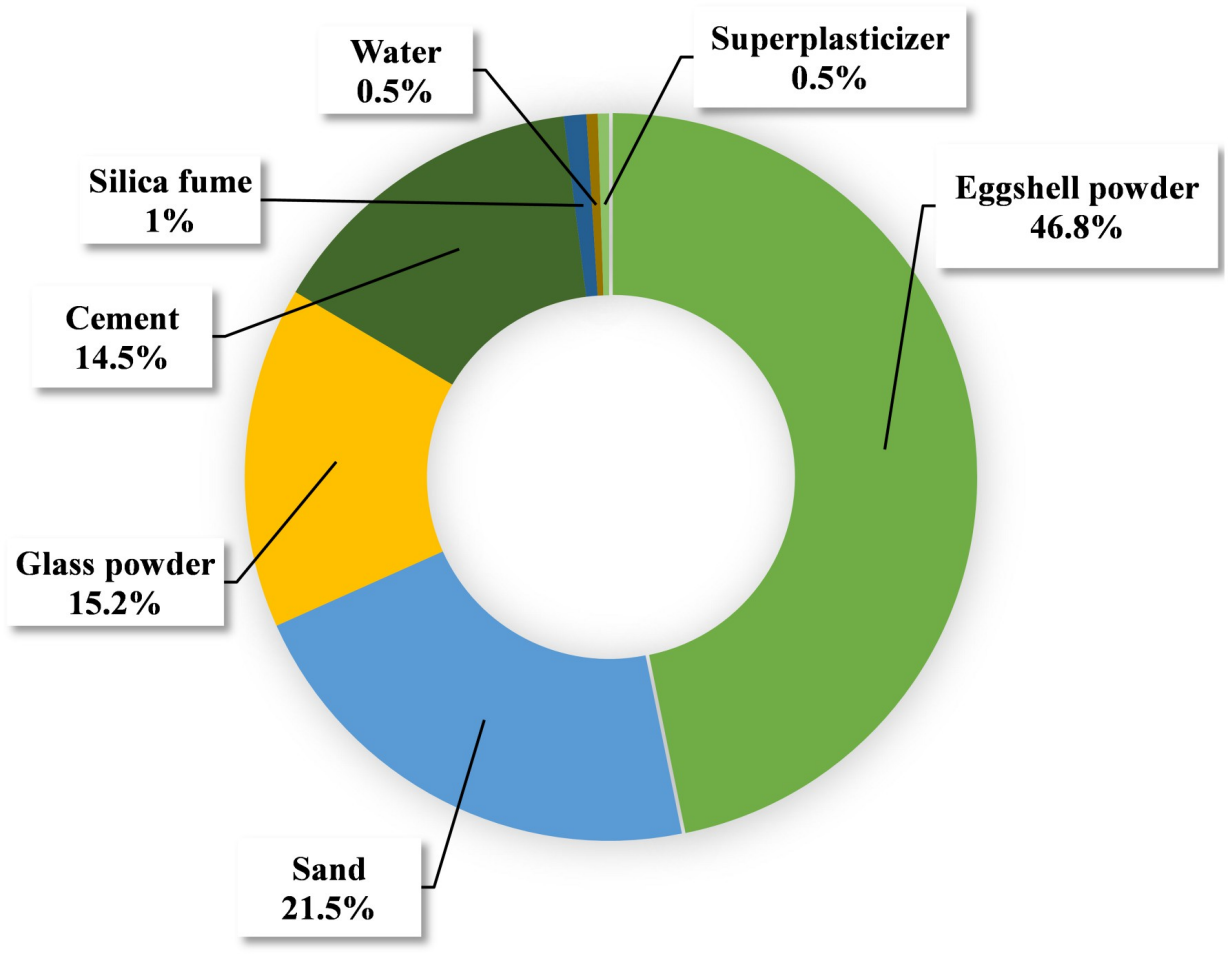
Sensitivity analysis of numerous contributing parameters towards the W.A prediction.

The purpose of this study is to examine how varying input parameters affect W.A prediction for E&GP-CM. There is a solid correlation between the input parameters and the predicted output [76]. The effect of each variable on the W.A. Fig 9 depicts a foresight into the future of mortar. The majority, 46.8%, came from eggshell powder, followed by sand (21.5%) and glass powder (15.2%). Cement had the most impact on predicting W.A of E&GP-CM, at 14.5%, followed by silica fume (1.0%), water (0.5%), and superplasticizer (0.5%). Results from sensitivity studies were proportional to the number of model parameters and data points included in the analysis. Mortar mix proportions and additional input parameters affected the analysis’s outcomes differently, although this was revealed by the ML technique’s application. Eqs (4) and (5) were used to determine the weighting of the model’s input variables:

$$N_{i}=f_{max\;}\left(x_{i}\right)\;-\;f_{min\;}\left(x_{i}\right)$$ (11)

$$S_{i}=\frac{N_{i}}{\sum_{j-i}^{n}N_{j}}$$ (12)

Where, f_max_ (x_i_) and f_min_ (x_i_) are the maximum and minimum expected values over the ith output, respectively.

#### 3.4.2 Scatter plots

**Fig 10:**
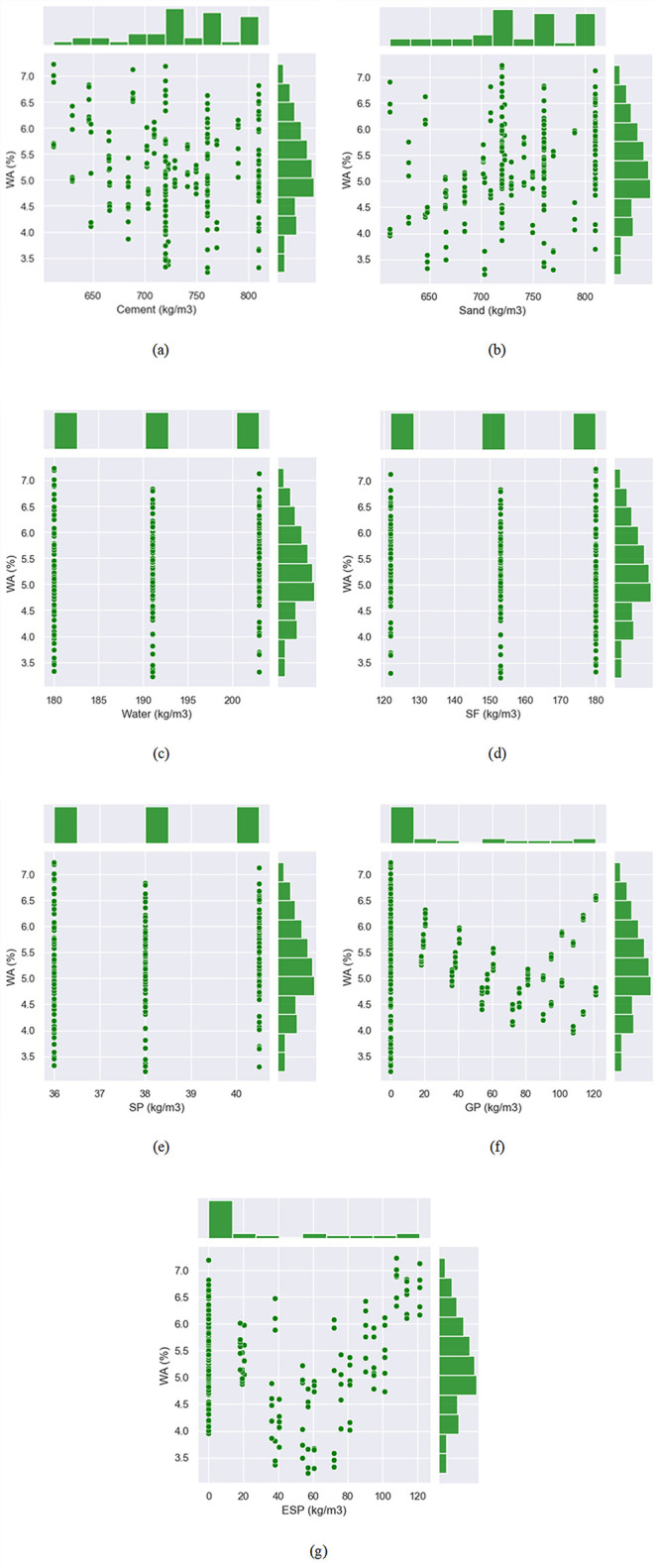
Interaction of input parameters with W.A of E&GP-CM: (a) Cement; (b) sand; (c) water; (d) silica fume (SF); (e) superplasticizer (SP); (f) glass powder (GP); (g) eggshell powder (EP).

Fig 10 depicts the raw material interaction required for the W.A of E&GP-CM. As can be seen in Fig 10(a), the cement amount interaction is diminishing; suggesting that including more cement in the mix decreases the W.A of E&GP-CM. Since sand interaction was shown to be growing (Fig 10(b)), it follows that adding more sand to the mixture could boost its W.A. Since there was less variation in these database values, as seen in Fig 10(c), 10(d) and 10(e), it was difficult to deduce the nature of the interactions between water, SP, and SF. Fig 10(f) displays a decreasing and, subsequently, increasing glass powder (GP) pattern, suggesting an ideal GP concentration of about 80 kg/m^3^. However, it was found that a lesser percentage of eggshell powder was optimal (about 60 kg/m^3^), suggesting that GP might be utilized in higher amounts than eggshell powder. Similar to the GP, it was found that the interaction of eggshell powder decreased and then increased (Fig 10(g)). The scatter plot indicates that EP and GP work best as sand replacements rather than cement substitutes, allowing for a smaller sand proportion and a larger cement quantity in the mix. The results of this study are directly attributable to the choice of independent variables and sample size. If more variables are included in the analysis, stronger associations may be discovered.

## 4 Discussion

**Fig 11:**
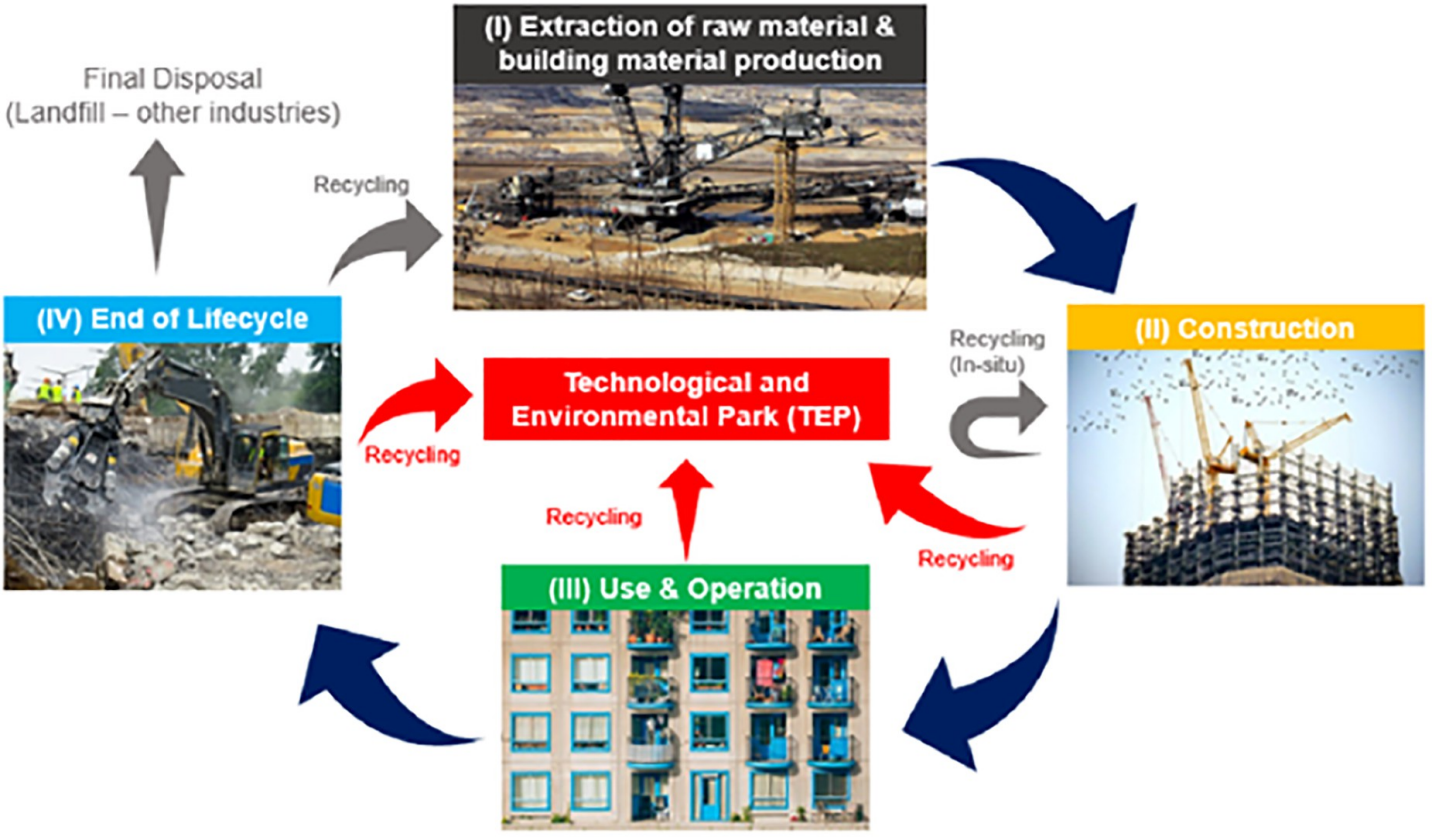
A model of circular economy by recycling waste in the construction sector [81,82].

The widespread use of regular Portland cement as the only binding material around the world depletes raw materials and accounts for around 5–8% of global anthropogenic emissions [77]. Because of this, the OPC sector must find environmentally preferable replacements for OPC in order to reduce its CO_2_ emissions. To address this issue, a wide variety of supplementary cementitious materials (SCM) have been used as partial replacements for cement/sand, with materials like fly ash, rice husk ash, silica fume, glass powder, eggshell powder, etc., proving to be among the most promising construction materials with low environmental impact and energy consumption [78]. Using recycled materials in the construction section will promote the circular economy, as depicted in Fig 11. Using ML and sensitivity analysis, this research aimed to deepen our familiarity with E&GP-CM (Eggshell Powder and Glass Powder-based Cement Mortar). In order to determine the W.A of E&GP-CM, this research utilized GEP and MEP ML techniques. The accuracy of each approach was evaluated to find the most reliable prediction. With an R^2^ of 0.90 for W, the MEP method provided more reliable results than the GEP method. A forecast based on the GEP-R^2^ of 0.88. Evidence of the MEP method’s higher precision was found in the discrepancy between actual and predicted results (errors). The error analysis demonstrates how well the experimental and predicted results of the MEP model accord when compared to the GEP models. For determining CBC strengths, the MEP approach has been demonstrated in prior research to be more accurate than the GEP ML method [79,80].

In addition, statistical methods were used to judge the efficacy of ML approaches. The smaller the deviation (MAE, RMSE, MAPE, etc.) and the higher the R^2^ value, the more accurate the model. Predicting attributes across different fields of study may be difficult, however, as algorithm effectiveness is heavily dependent on the quantity of inputs and data samples employed. The predictions of the two models were compared using several different error metrics, and it was determined that MEP was more accurate than GEP. The impact of different raw materials on the W.A of E&GP-CM was further investigated by sensitivity analysis and interaction scatter plots. Cement, eggshell powder, glass powder, and sand were all shown to have a significant correlation with the final output of W.A., demonstrating their usefulness as input features. Water, superplasticizer, and SF had less clear effects as the data variance shrank. Therefore, it can be concluded that partially replacing OPC with eggshell and glass powder in CBCs will result in superior construction material with comparatively equal performance in terms of strength. It will also aid in the critically important task of managing the limited stocks of raw materials required in OPC production.

## 5 Conclusions

Machine learning (ML) models examined how eggshell powder (E) and recycled glass powder (GP) in cement mortar (E&GP-CM) affected water absorption (W.A). Two ML models, Gene Expression Programming (GEP) and Multi Expression Programming (MEP), predicted E&GP-CM’ W.A utilizing experiment data. The research’s main findings:

- The GEP approach was accurate enough for W.A estimation of E&GP-CM (R^2^ = 0.88), but the MEP method was more precise (R^2^ = 0.90).
- The average gap between experimental and projected W.A (errors) in GEP and MEP techniques was 0.2528% and 0.1075%, respectively. These error rates also showed that the GEP model was correct, and that the MEP technique forecasted E&GP-CM W.A more accurately.
- Statistical validation has proven the effectiveness of the models used. ML models have improved R^2^ and reduced errors. The GEP model had 5.20% MAPE and the MEP model 3.30%. The MEP model had 0.107% MAE, and the GEP model 0.253%. These judgments supported other areas of model performance validation.
- Sensitivity study shows that the E amount has the greatest impact on W.A of E&GP-CM, followed by sand, GP, cement, silica fume, water, and superplasticizer (respectively 46.8%, 21.5%, 15.2%, 14.5%, 1%, 0.5%, and 0.5%).
- The ideal E and GP levels in the mix were 60 kg/m3 and 80 kg/m3, respectively, according to raw material interaction study. The W.A of E&GP-CM was favorably connected with sand quantity but negatively correlated with cement quantity, suggesting that replacing sand with E and GP is better than replacing cement.

The W.A of E&GP-CM may be estimated using the ML-based prediction models constructed with varied amounts of inputs, including cement, water, sand, silica fume, superplasticizer, recycled glass powder, and eggshell powder. Other factors, including the water-binder ratio, the chemical and physical properties of components, and environmental factors, may also affect the W.A of E&GP-CM. Therefore, more research is needed to compile a more extensive database with a greater variety of input parameters for creating ML-based prediction models.

## Supporting information

S1 Data (CSV)
